# A strategy for QTL fine-mapping using a dense SNP map

**DOI:** 10.1186/1753-6561-3-s1-s3

**Published:** 2009-02-23

**Authors:** Joaquim Tarres, François Guillaume, Sébastien Fritz

**Affiliations:** 1 UR337 Station de Génétique Quantitative et Appliquée, INRA, Jouy-en-Josas F-78350, France; 2 Institut de l'élevage, Paris F-75595, France; 3 Union Nationale des Coopératives agricoles d'Elevage et d'Insémination Animale, Paris F-75595, France

## Abstract

**Background:**

Dense marker maps require efficient statistical methods for QTL fine mapping that work fast and efficiently with a large number of markers. In this study, the simulated dataset for the XIIth QTLMAS workshop was analyzed using a QTL fine mapping set of tools.

**Methods:**

The QTL fine-mapping strategy was based on the use of statistical methods combining linkage and linkage disequilibrium analysis. Variance component based linkage analysis provided confidence intervals for the QTL. Within these regions, two additional analyses combining both linkage analysis and linkage disequilibrium information were applied. The first method estimated identity-by-descent probabilities among base haplotypes that were used to group them in different clusters. The second method constructed haplotype groups based on identity-by-state probabilities.

**Results:**

Two QTL explaining 9.4 and 3.3% of the genetic variance were found with high significance on chromosome 1 at positions 19.5 and 76.6 cM. On chromosome 2, two QTL were also detected at positions 26.0 and 53.2 explaining respectively 9.0 and 7.8 of total genetic variance. The QTL detected on chromosome 3 at position 11.9 cM (5% of variance) was less important. The QTL with the highest effect (37% of variance) was detected on chromosome 4 at position 3.1 cM and another QTL (13.6% of variance) was detected on chromosome 5 at position 93.9 cM.

**Conclusion:**

The proposed strategy for fine-mapping of QTL combining linkage and linkage disequilibrium analysis allowed detecting the most important QTL with an additive effect in a short period but it should be extended in the future in order to fine-map linked and epistatic QTL.

## Background

High-throughput SNP analysis and SNP micro-arrays now give the opportunity to genotype many animals for hundreds of SNP per chromosome. Thanks to these techniques, marker density is no longer a limiting factor in QTL fine-mapping studies. However, these dense marker maps require statistical methods that work fast and efficiently with a large number of markers.

The purpose of this paper was to present a strategy for QTL fine-mapping and its corresponding results on the XIIth QTLMAS workshop simulated dataset.

## Methods

The QTL fine-mapping strategy was mostly based on the use of statistical methods combining linkage (LA) and linkage disequilibrium analysis (LDLA) described by Druet et al. [1].

### Linkage analysis

First, a variance component-based (VC) linkage analysis [2] was performed at each marker position with the following model:

**y **= **μ **+ **Zu **+ **Z**_**v**_**v **+ **e**

where **y **is a vector containing the phenotypic values for bulls, **μ **is the mean, **u **is a vector of random polygenic effects, **v **is a vector of random gametic effects and **e **is a vector of random residual terms. **Z **and **Z**_**v **_are known design matrices relating the results to random polygenic and gametic effects, respectively.

The (co)variance structure was:

$$\mathrm{var}\left[\begin{array}{c} u\\ v\\ e \end{array}\right]=\left[\begin{array}{ccc} A\sigma_{u}^{2} & 0 & 0\\ 0 & G_{v}\sigma_{v}^{2} & 0\\ 0 & 0 & R \end{array}\right]$$

where **R **is a diagonal matrix containing the residual variance ($\sigma_{e}^{2}$). **A **is the additive relationship matrix and $\sigma_{u}^{2}$ is the polygenic variance. **G**_**v **_is the relationship matrix among QTL allelic effects estimated due to relationships and marker information [3] and $\sigma_{v}^{2}$ is the gametic variance. As in Pong-Wong et al. [4], the method for calculating the gametic matrix used the closest informative bracket instead of estimating probabilities-of-descent of a gamete (PDQ) from parent to offspring by integration over all possible haplotypes. Rules to compute the PDQ using the closest informative bracket can be found in Table 1 in Pong-Wong et al. [4]. The variances of paternal and maternal alleles were assumed to be equal and a single parameter was estimated ($\sigma_{v}^{2}$). Then, variance associated to the QTL (QTL allelic variance) was twice $\sigma_{v}^{2}$. The proportion of total genetic variance due to the QTL was

$$\frac{2\sigma_{v}^{2}}{\sigma_{u}^{2}+2\sigma_{v}^{2}}.$$

**Table 1:** Position (and confidence interval) of the QTL inferred in the different chromosomes with LA, LDLA HAP3 and LDLA IBD10 models and percentage of genetic variance explained by the QTL at the selected position

Chromosome	LA	HAP3	IBD10	Selected position	% genetic variance
1	20.9 (14.2–26.3)	19.5 (18.1–20.2)	19.5 (18.2–20.3)	19.5	9.4
	76.6 (73.4–78.1)			76.6	3.3
2	29.0 (22.4–36.1)	31.0 (25.4–31.6)	26.0 (25.4–29.2)	26.0	9.0
	53.2 (51.5–55.3)			53.2	7.8
3	11.8 (11.3–15.6)	11.9 (11.3–14.1)	11.9 (11.0–15.0)	11.9	5.0
4	3.9 (0.6–12.0)	3.1 (2.8–5.0)	4.8 (2.0–5.7)	3.1	37.0
5	96.1 (90.2–98.2)	93.9 (92.9–94.0)	93.5 (92.7–94.6)	93.9	13.6

Genetic parameters were estimated after maximizing likelihoods with an AI-REML approach. The BLUPF90 software [5] was modified by Druet et al. [1] to incorporate relationship matrices among QTL allelic effects.

The likelihood ratio test statistic considered variance components as parameters and was used to confirm whether there was a QTL present at the studied position [2]:

$$\lambda =-2\ln\frac{L(H_{0})}{L(H_{1})}$$

where *L*(*H*_0_) and *L*(*H*_1_) are the values of the likelihood functions estimated by REML under the polygenic model with no QTL fitted and the model with QTL respectively. The distribution of the test is a mixture of zero and 1-d.f. chi-square for a single position [6]. For the analyses where a significant QTL was detected, a 2-LOD-dropoff support interval was constructed for the position of the QTL, i.e., the interval surrounding the QTL peak where the likelihood exceeds ln *L*_max _-2ln(10), where ln *L*_max _is the natural logarithm of the maximum likelihood [7].

### Combined linkage disequilibrium and linkage analysis (LDLA)

Linkage analysis gives an interval region for the QTL. Within this region, QTL fine mapping with LDLA was applied based on an approach derived from the method proposed by Meuwissen and Goddard [8]. It consists of a VC mapping method that includes information from linkage disequilibrium between base haplotypes in the construction of the relationship matrix among estimated QTL allelic effects (see above). Chromosomes of founders were considered as base haplotypes. At each tested position the following procedure was applied:

1. PDQ probabilities were computed to determine to which base haplotype an inherited chromosome corresponded. Rules to compute the PDQ using the closest informative bracket [4] were the same as those used in linkage analysis. LD information was not taken into account at this step.

2. Identity-by-descent (IBD) probabilities (Φ_*p*_) were estimated among each pair of base haplotypes conditionally on the identity-by-state (IBS) status of the neighboring markers using windows of 10 flanking markers [7].

3. Base haplotypes were grouped with a clustering algorithm with SAS^® ^proc CLUST using (1-Φ_*p*_) as a distance measure. Base haplotypes were grouped if Φ_*p *_exceeded 0.50 [1]. Indeed, Ytournel (personal communication) showed that most haplotypes were IBD as soon as their estimated IBD probability exceeded 0.5. Chromosomes were also grouped within the clusters if i) the two chromosomes of a sire were grouped in the same cluster (the paternally inherited chromosomes of all his sons were then grouped in this cluster) or ii) a chromosome could be associated to a base haplotype with a probability larger than 0.95 (it was grouped to the corresponding cluster).

4. A model similar to the linkage analysis model was then applied:

**y **= **μ **+ **Zu **+ **Z**_**h**_**h **+ **e**

where h is a vector of random QTL effects corresponding to the haplotype clusters and **Z**_**h **_is a design matrix relating phenotypes to corresponding haplotype clusters. IBD10 will be the notation for this model.

In addition, a similar model with the following new rules was applied: 1) all chromosomes were considered as base haplotypes; 2) smaller marker windows were used (3 markers) in order to obtain a limited number of clusters and 3) haplotype groups were no longer constructed based on IBD probabilities but on IBS status (if haplotypes were IBS for all markers they were grouped together). This method evaluates whether an effect can be associated to a small haplotype covering a small region. HAP3 will be used to refer to this model.

## Results

The analysis was performed on a 64-bit IBM AIX 5.2.0 server with power4+ processor and 62 Gb RAM. The LA, IBD10 and HAP3 methods needed respectively 16.4, 17.2 and 445 seconds of CPU time for each marker. These numbers should be multiplied by the number of marker positions tested. Here, it is important to note that marker information for the first two generations was discarded in our study in order to reduce the total time required for the analysis. Haplotypes for these animals were reconstructed by working with dense marker maps using a program developed by Druet et al. [1].

**Figure 1:**
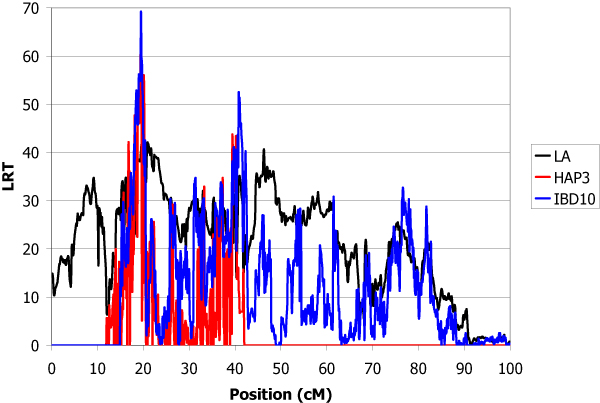
**LA and LDLA curves obtained on chromosome 1**. LA curve (black), LDLA curve with model HAP3 (red) and LDLA curve with model IBD10 (blue).

**Figure 2:**
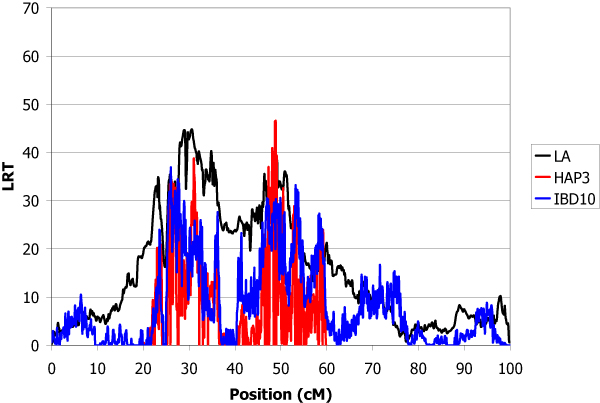
**LA and LDLA curves obtained on chromosome 2**. LA curve (black), LDLA curve with model HAP3 (red) and LDLA curve with model IBD10 (blue).

**Figure 3:**
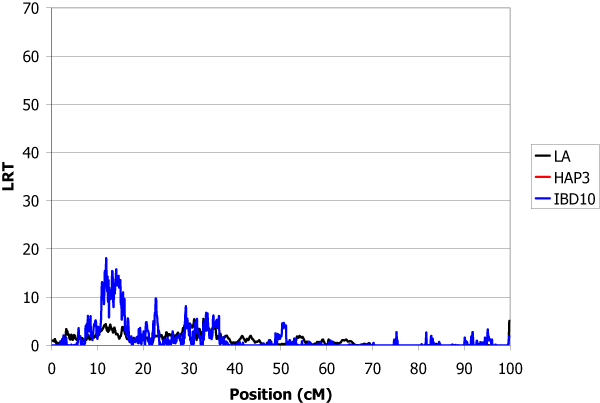
**LA and LDLA curves obtained on chromosome 3**. LA curve (black), LDLA curve with model HAP3 (red) and LDLA curve with model IBD10 (blue).

**Figure 4:**
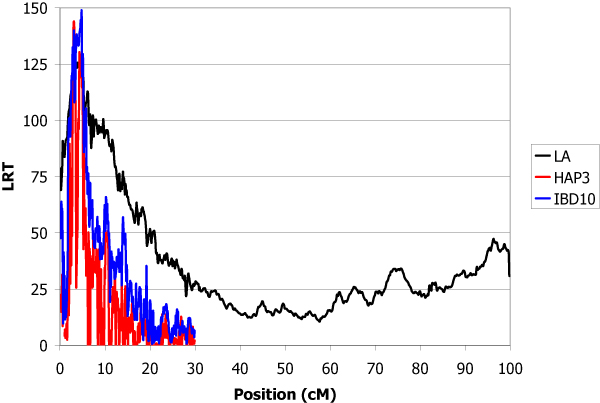
**LA and LDLA curves obtained on chromosome 4**. LA curve (black), LDLA curve with model HAP3 (red) and LDLA curve with model IBD10 (blue).

**Figure 5:**
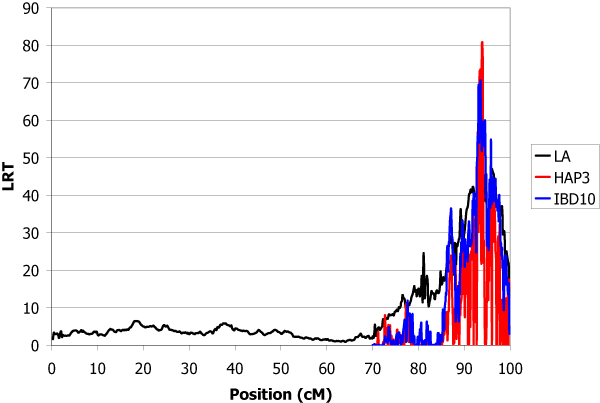
**LA and LDLA curves obtained on chromosome 5**. LA curve (black), LDLA curve with model HAP3 (red) and LDLA curve with model IBD10 (blue).

The estimated total genetic variance of the trait was 1.32 and the heritability was 0.30. The locations of inferred QTL using the LA, IBD10 and HAP3 methods are shown in Table 1. IBD10 and HAP3 methods give several peaks with LRT higher than for linkage analysis. The use of the haplotypes of heterozygous sires at the QTL offered the possibility to give confidence to some of them. A QTL explaining 9.4% of the genetic variance was found with high significance in chromosome 1 at position 19.5 cM (Figure 1). In chromosome 2, the main QTL was detected at position 26.0 cM which explained 9.0% of total genetic variance (Figure 2). The QTL detected in chromosome 3 at position 11.9 cM was less important (5% of variance) (Figure 3). The QTL with the highest effect (37% of variance) was detected in chromosome 4 at position 3.1 cM (Figure 4) and another QTL (13.6% of variance) was detected in chromosome 5 at position 93.9 cM (Figure 5). No QTL was detected in chromosome 6.

These results were based on a model assuming a single QTL per chromosome. However, in order to test whether there was another QTL present in a chromosome, the LA model was extended with a second QTL effect. This model allowed finding a second QTL with high significance at positions 76.6 cM in chromosome 1 and 53.2 cM in chromosome 2 explaining respectively 3.3 and 7.8% of the genetic variance.

## Discussion

Our QTL fine-mapping strategy was mostly based on the use of statistical methods combining linkage (LA) and linkage disequilibrium analysis (LDLA) described by Druet et al. [1]. Linkage analysis provides a LRT peak and a confidence interval for the location of the QTL in each chromosome. The use of a high density marker map resulted in almost optimal genetic information along the whole chromosome. In consequence, sharp and high LRT curves were obtained. With this density of markers, QTL transmission is followed more precisely and locations of recombinations are determined within smaller intervals allowing an almost perfect achievement of the pedigree linkage mapping resolution. The method used for LDLA analysis was based on LDLA methods proposed by Meuwissen and Goddard [7]. Despite the fact that the LDLA analysis did not result in a single peak, it improved strongly the information on the QTL location with respect to the LA analysis. Indeed, many regions could be discarded according to the LDLA analysis because QTL alleles of opposite effects were grouped in the same cluster. The LDLA analysis discarded regions where heterozygous sires did not share common haplotypes. As a consequence, the possible location of the QTL is confined to a few small intervals. The HAP3 and IBD10 models have some complementary properties. First, HAP3 searches for small informative regions of 3 markers in LD with the QTL. The IBD10 method uses IBD probabilities and uses a large marker window. Therefore, it helps to discard regions that were identical for three markers by chance from regions where haplotypes were grouped because they have high IBD probabilities. However, IBD10 will be more sensitive to missing information or to genetic map inconsistencies.

This strategy was thought to detect the most important QTL with an additive effect for an important number of traits in a relatively short time period. The advantage of our strategy was that it allows reducing the number of regions to be analyzed using LDLA methods which provided sharper and higher LRT peaks than other available methods. It will be applied to the analysis of 60,000 SNP data of 3300 bulls for 15 traits from May 2008 to July 2008. The aim of this analysis is to select 1,500 SNP in LD with QTL so they can be used for routine marker assisted selection (MAS). In this simulation study, the position of the main simulated QTL from each chromosome was correctly estimated, in spite of its effect (measured as a proportion of genetic variance) was sometimes over or under estimated. Alternatively, in the chromosomes where several QTL located in different positions were affecting the trait, fine-mapping of the linked QTL using linkage analysis was not very efficient and more complex methods such as multi-QTL LDLA fine-mapping methods [9,10] are needed. The programs should also be extended in order to detect possible epistatic loci.

## Conclusion

The proposed strategy for fine-mapping of QTL using a dense SNP map worked relatively fast with a large number of markers. The linkage analysis approach provides a confidence interval for the QTL. Within these intervals, the QTL position is fine-mapped applying two variance component approaches combining both linkage analysis and linkage disequilibrium information. This strategy allows detecting the most important QTL with an additive effect for an important number of traits in a short time period but it should be extended for fine-mapping linked and epistatic QTL.

## List of abbreviations used

IBD: Identity-by-descent; IBS: Identity-by-status; LA: Linkage analysis; LD: Linkage disequilibrium; LDLA: Linkage disequilibrium and linkage analysis; QTL: Quantitative trait loci; SNP: Single nucleotide polymorphism; VC: Variance components.

## Competing interests

The authors declare that they have no competing interests.

## Authors' contributions

JT: Performed the statistical analysis and drafted the manuscript. FG: Performed the statistical analysis. SF: Performed the statistical analysis. All authors read and approved the final manuscript.
